# A Crisscross-Enhanced Groupers and Moray Eels Optimization Algorithm: Benchmark Test and Production Optimization

**DOI:** 10.3390/biomimetics11050322

**Published:** 2026-05-06

**Authors:** Yuwei Fan, Zhilin Cheng, Youyou Cheng

**Affiliations:** 1 Changqing Drilling Company, CNPC Chuanqing Drilling Engineering Company Limited, Xi’an 710018, China; zj1bfyw@cnpc.com.cn; 2 Engineering Research Center of Development and Management for Low to Ultra-Low Permeability Oil & Gas Reservoirs in West China, Ministry of Education, Xi’an 710065, China; 3 College of Petroleum Engineering, Xi’an Shiyou University, Xi’an 710065, China; 4 School of Earth Sciences and Engineering, Xi’an Shiyou University, Xi’an 710065, China; yycheng@xsyu.edu.cn

**Keywords:** Groupers and Moray Eels Optimization, crisscross strategy, global optimization, metaheuristic algorithms, HCS, Vertical Crossover Search, benchmark functions

## Abstract

Metaheuristic algorithms can fail to balance global exploration and local exploitation, occasionally becoming trapped in suboptimal regions on highly multimodal problems. The Groupers and Moray Eels (GME) algorithm, inspired by the associative hunting strategies of marine predators, provides a cooperative optimization framework. However, the sequential interaction phases of GME can fail to maintain diverse topological coverage across heavily constrained landscapes. To address these limitations, we propose an enhanced variant, GPS-CC-GME. The approach improves the initial agent distribution by deploying a number-theoretic Good Point Set (GPS) generation protocol to establish a uniformly dispersed starting space. In addition, algorithmic stagnation is addressed through a dual-crossover search architecture. A horizontal crossover stage enforces information sharing among randomized agents to sustain global diversity, and a vertical crossover phase isolates specific dimensional vectors within individual agents for localized fine-tuning. We evaluated the proposed model on the CEC2017 benchmark suite, where it secured the highest overall ranking compared to the baseline GME and several standard metaheuristics. GPS-CC-GME was then applied to a high-dimensional optimization scenario for petroleum reservoir production. The algorithm yielded higher Net Present Value (NPV) metrics than the canonical framework. The results indicate that embedding deterministic initialization and bidirectional mutation operators into multipredator models can improve search outcomes in non-linear engineering tasks.

## 1. Introduction

Optimization methodologies are used to identify optimal solutions from large sets of alternatives [1,2]. This process aims to improve efficiency and resource allocation in various engineering and economic applications [3,4,5,6]. Real-world problems are frequently characterized by high-dimensional, nonlinear, and multimodal landscapes that pose computational challenges, requiring advanced solution strategies [7,8,9,10].

Traditional optimization methods, including gradient-based techniques and the simplex algorithm [11,12,13], are typically used for well-behaved, convex problems. They are generally efficient for local search and converge reliably when the objective function is smooth, continuous, and unimodal [14]. However, gradient-based approaches are highly sensitive to initial conditions and frequently converge to local minima in nonconvex settings [15]. Similarly, enumerative methods become computationally intractable because the computational cost grows exponentially with problem size [16]. These limitations motivate the development of optimization strategies capable of navigating discontinuous, high-dimensional search spaces.

Metaheuristic algorithms are alternatives that do not share the limitations of classical methods [17]. Metaheuristics require fewer assumptions about problem structure and do not need gradient information or convexity [18]. This independence allows them to handle complex, non-differentiable, and noisy objective functions [19]. A principal advantage is their global search capability; they use stochastic strategies to explore the search space, reducing susceptibility to local optima [20]. Using mechanisms inspired by biological evolution, swarm intelligence, or physical processes, metaheuristics provide frameworks for identifying high-quality solutions [21].

Metaheuristic algorithms are broadly categorized into evolutionary algorithms (EAs) and swarm intelligence (SI) algorithms [17]. EAs emulate natural selection and genetics through mechanisms such as mutation, crossover, and selection. Examples include Genetic Algorithms (GAs) [22,23] and Differential Evolution (DE) [24]. SI algorithms emulate the collective behavior of decentralized systems, such as bird flocks and ant colonies [25]. These methods rely on social interaction to guide the search process, with instances including Particle Swarm Optimization (PSO) [26] and Ant Colony Optimization (ACO) [27].

Despite their success, metaheuristic algorithms are subject to the No Free Lunch (NFL) theorem [28], which states that no single algorithm is universally superior across all problem domains. Therefore, an algorithm that excels in one problem class may perform poorly in another, motivating the ongoing development of novel and hybrid algorithms [18,29]. Practical challenges include premature convergence to local optima, computational cost, and problem-specific parameter tuning [30]. These limitations motivate the development of adaptive metaheuristics for complex applications [31]. Recent advancements have introduced highly competitive algorithms such as L-SHADE, jSO, RIME, and INFO, which broaden the scope of applications to diverse engineering domains.

This study adopts the Groupers and Moray Eels (GME) algorithm as a foundational framework [32]. GME models the associative hunting behavior between groupers and moray eels. In nature, groupers rely on speed in open water, while moray eels navigate narrow spaces. The algorithm uses a four-phase mechanism: primary search, pair association, encircling, and attacking. Although GME balances exploration and exploitation, it has structural limitations that can degrade performance in complex landscapes. The algorithm depends on fixed movement vectors during pair association and encircling. This topology limits broad horizontal information exchange across the population, which can lead to premature convergence on multimodal problems. Additionally, the standard pseudo-random initialization procedure can lead to uneven spatial coverage, which may be unsuited for high-dimensional search spaces.

To address these limitations, we propose GPS-CC-GME, which integrates a crisscross (CC) strategy and deterministic initialization into GME. The method uses a Good Point Set (GPS) sequence to ensure uniform initial coverage of the search space. The CC mechanism incorporates two operations: Horizontal Crossover Search (HCS), which exchanges information between distinct agents to aid global exploration, and Vertical Crossover Search (VCS), which performs dimension-wise positional refinement within individuals to aid local exploitation. This integration preserves population diversity and accelerates convergence.

The main contributions of this work include the development of the GPS-CC-GME algorithm, which integrates GPS initialization and the CC strategy to address the diversity limitations of GME. Horizontal and vertical crossover searches are employed to improve population coverage and convergence. The global optimization capability of the algorithm is then evaluated against nine metaheuristic algorithms on the CEC2017 benchmark suite, supported by Wilcoxon signed-rank and Friedman statistical tests. Finally, GPS-CC-GME is applied to a 75-variable reservoir production optimization problem, yielding a higher NPV than the compared algorithms and demonstrating its utility in complex engineering scenarios.

The remainder of this paper is organized as follows. Section 2 outlines the original GME algorithm. Section 3 details the proposed GPS-CC-GME algorithm, covering the GPS sequence and the CC strategy. Section 4 describes the experimental setup and the comparative results on benchmark functions. Section 5 presents the application of GPS-CC-GME to petroleum reservoir production optimization. Section 6 concludes the paper.

## 2. Original Algorithm Overview: GME Optimization

GME, proposed by Mansour et al. [32], is a swarm intelligence algorithm inspired by the associative hunting behavior of groupers and moray eels in coral reef ecosystems. Unlike cooperative hunting among conspecifics, associative hunting involves distinct species leveraging complementary skills to capture prey. The grouper uses its speed to hunt in open waters and signals the moray eel to flush out prey hidden in narrow crevices. GME numerically translates this multipredator strategy to maintain a delicate balance between exploration and exploitation. The generic GME optimization process consists of four distinct phases: primary search, pair association, encircling, and attacking.

### 2.1. Mechanisms

#### 2.1.1. Primary Search (PS) Phase

The PS phase models groupers scouting for prey using a zig-zag swimming pattern. Let *N* be the population size, divided equally into groupers (*G*) and moray eels (*E*). The initialization of groupers within the boundaries $\left[lb_{j},ub_{j}\right]$ is formulated as:

(1) $$X_{i,j}^{1}=lb_{j}+r\cdot \left(ub_{j}-lb_{j}\right),\;i=1,2,\ldots ,N/2;j=1,2,\ldots ,D$$

where *r* is a random scalar uniformly distributed in [0,1]. During the PS iterations, groupers move randomly in successive hops. The updated position expands toward the maximum boundary on even hops and moves inward on odd hops. At the end of the PS phase, the best position identified by each grouper serves as its tracking vector.

#### 2.1.2. Pair Association (PA) Phase

In the PA phase, each grouper is paired with a distinct moray eel. The pairing is based on objective fitness, with groupers and eels sorted in descending order. The highest-ranked grouper is paired with the highest-ranked eel.

#### 2.1.3. Encircling or Extended Search (ES) Phase

In the ES phase, both predators encircle the target location. The coordinates of the prey are interpolated between the grouper and the eel. The grouper approaches using a logarithmic spiral, and the moray eel approaches using a sinusoidal wave. For a grouper *i* at iteration *t*, the position update vector $X_{i}^{t+1}$ is computed as:

(2) $$X_{i}^{t+1}=D_{1}\cdot e^{kw}\cos\left(2\pi w\right)+X_{best}^{t}$$

where $D_{1}$ is the distance vector to the prey, *k* defines the spiral shape, *w* linearly decreases with the iteration count, and $X_{best}^{t}$ is the global best position at iteration *t*. The position of the moray eel $X_{i}^{t+1}$ is updated using:

(3) $$X_{i}^{t+1}=\alpha \cdot \lambda^{t}\cdot \xi \cdot \sin\left(g\right)+X_{i}^{t}$$

where $\lambda^{t}$ signifies the directional wavelength distance at iteration *t*, α is a random scalar uniformly distributed in [0,1], and ξ defines the angular wave amplitude.

#### 2.1.4. Attacking and Catching (AC) Phase

The AC phase forms a multidimensional spherical boundary around the global best location. The radius *R* of this boundary is reduced at each iteration *t* using a shrinking factor μ:

(4) $$R^{t+1}=\left(1-\mu \right)\cdot R^{t}$$

Agents are stochastically repositioned within this shrinking hypersphere until termination.

### 2.2. Integrated Iteration

The GME algorithm initializes agents, evaluates fitness, and partitions them into groupers and moray eels. The optimization loop then executes four stages. The PS phase uses zig-zag movements. The PA phase pairs agents based on fitness. These pairs execute the ES phase, encircling targets using logarithmic and sinusoidal trajectories. Finally, the AC phase confines the population within a contracting hypersphere. The cycle repeats until the computational budget is exhausted.

## 3. Proposed Algorithm: GPS-CC-GME

GPS-CC-GME integrates a deterministic initialization method and a dual-crossover search strategy into the GME framework. These mechanisms address limitations related to initial population distribution and search diversity. The algorithm uses GPS Initialization and the CC Strategy, which consists of HCS and VCS operators.

### 3.1. GPS Initialization

GPS initialization replaces standard pseudo-random initialization. Based on number theory, GPS uses a low-discrepancy sequence to distribute population points uniformly across the search space. This approach ensures initial spatial coverage and reduces the risk of early clustering.

Mathematical Formulation: For a population size *N* and problem dimension *D*, the initial spatial position for the *i*-th individual in the *j*-th dimension is computed as:

(5) $$X_{i,j}^{1}=lb_{j}+\left(ub_{j}-lb_{j}\right)\cdot \left\{r_{j}\cdot i\right\}$$

where {·} denotes the fractional part operator, $X_{i,j}^{1}$ is the initialized position of the *i*-th individual in the *j*-th dimension at iteration t=1, and $lb_{j}$ and $ub_{j}$ are the boundary constraints of the *j*-th dimension. The term $r_{j}$ is the *j*-th component of a pre-constructed vector r, formatted as $r_{j}=\left\{2\cos\left(2\pi j/p\right)\right\}$, where *p* is the smallest prime number satisfying p≥2D+1.

### 3.2. CC Strategy: HCS

The HCS operator facilitates information exchange between distinct individuals. HCS performs a dimension-wise crossover between randomly paired individuals. For any randomly selected pair of distinct individuals, $X_{i}^{t}$ and $X_{k}^{t}$, the horizontal crossover is performed across every dimension *j* to generate offspring:

(6) $$H_{i,j}^{t}=r_{1}\cdot X_{i,j}^{t}+\left(1-r_{1}\right)\cdot X_{k,j}^{t}+c_{1}\cdot \left(X_{i,j}^{t}-X_{k,j}^{t}\right)$$

(7) $$H_{k,j}^{t}=r_{2}\cdot X_{k,j}^{t}+\left(1-r_{2}\right)\cdot X_{i,j}^{t}+c_{2}\cdot \left(X_{k,j}^{t}-X_{i,j}^{t}\right)$$

where $X_{i,j}^{t}$ and $X_{k,j}^{t}$ denote the *j*-th dimensional values of individuals *i* and *k* at iteration *t*, $H_{i,j}^{t}$ and $H_{k,j}^{t}$ are the corresponding offspring values, $r_{1}$ and $r_{2}$ are independent random variables uniformly distributed in [0,1], and $c_{1}$ and $c_{2}$ are random scaling coefficients distributed in [−1,1].

### 3.3. CC Strategy: VCS

The VCS operator performs intra-individual interaction between different dimensions of a candidate solution. VCS recombines internal dimensional vectors to refine the solution. For a given individual *i* at iteration *t*, two distinct dimensions ($j_{1}$ and $j_{2}$) are randomly selected, and a new positional coordinate is computed as:

(8) $$V_{i,j_{1}}^{t}=r_{3}\cdot X_{i,j_{1}}^{t}+\left(1-r_{3}\right)\cdot X_{i,j_{2}}^{t}$$

where $X_{i,j_{1}}^{t}$ and $X_{i,j_{2}}^{t}$ are the positional values of individual *i* in dimensions $j_{1}$ and $j_{2}$, $V_{i,j_{1}}^{t}$ is the newly generated coordinate for dimension $j_{1}$, and $r_{3}$ is a random variable sampled uniformly from [0,1].

To govern the execution sequences of these crossover operations, the overarching CC strategy undergoes a strict probabilistic evaluation during each main iteration. A uniformly distributed random variable r∼U(0,1) is independently sampled and compared against the predefined activation probability threshold $P_{cc}$. The combined crisscross mechanism is exclusively launched if $r\leq P_{cc}$. Upon generating prospective offspring via horizontal and vertical pathways, a strict greedy selection mechanism is mathematically applied to determine survival:

(9) $$X_{i}^{t+1}=\left\{\begin{array}{cc} H_{i}^{t}, & \mathrm{if}\;f\left(H_{i}^{t}\right)<f\left(X_{i}^{t}\right)\\ X_{i}^{t}, & \mathrm{otherwise} \end{array}\right.$$

Likewise, for the VCS operation, the candidate vector $V_{i}^{t}$ (which assimilates the newly modified $V_{i,j_{1}}^{t}$ coordinate) explicitly overwrites the parental vector $X_{i}^{t}$ merely under the condition $f\left(V_{i}^{t}\right)<f\left(X_{i}^{t}\right)$. These mathematical constraints directly preserve elite traits whilst preventing objective regression. Key parameters, including $P_{cc}$ and the population size *N*, were determined based on empirical guidelines and calibrated through preliminary experiments. The integration of HCS and VCS balances global exploration and local exploitation. Biomimetically, the HCS operator models interaction between the species (grouper and moray eel) during associative hunting, facilitating lateral information exchange. The VCS operator models localized behavioral refinements of a single predator, enforcing dimension-wise precision.

### 3.4. Overall Framework of GPS-CC-GME

**Figure 1:**
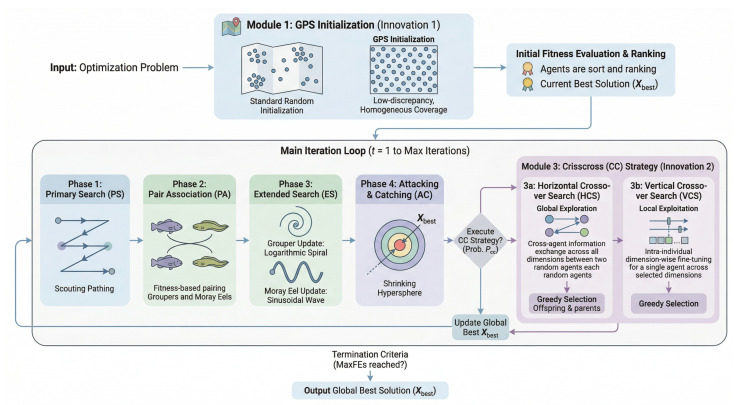
Overall framework of the proposed GPS-CC-GME algorithm.

The new mechanisms are embedded into the canonical GME workflow (Figure 1). The GPS initialization replaces the original random initialization of groupers and eels. The CC strategy is incorporated as an additional, probabilistically triggered search phase after the attacking and catching (AC) step. The algorithm steps are detailed in Algorithm 1.
**Algorithm 1** GPS-CC-GME Algorithm Framework  1:**Initialization:** Generate initial population *X* using GPS method;  2:Evaluate fitness for all individuals in *X*;  3:Determine global best $X_{best}$;  4:**while** termination criteria not met **do**  5:      **Primary Search (PS):** Groupers survey with zig-zag movement;  6:      **Pair Association (PA):** Groupers and eels form pairs based on fitness;  7:      **Encircling Search (ES):** Pairs encircle prey via spiral and sinusoidal paths;  8:      **Attacking & Catching (AC):** Shrink spherical boundary to capture prey;  9:      **if** $rand\left(\right)<P_{cc}$ **then**10:            **HCS:** Perform crossover between random pairs;11:            **VCS:** Perform crossover between dimensions;12:            Update individuals if fitness improves (Greedy Selection);13:      **end if**14:      **Consolidation:** Update global best solution $X_{best}$;15:**end while**16:**return** Best solution $X_{best}$;

The overall computational complexity of GPS-CC-GME depends on population size (*N*), problem dimensionality (*D*), and maximum iterations (*T*). GPS initialization requires O(ND) operations. Within each iteration, the four GME phases require O(ND) operations. The CC strategy, when triggered, incurs an additional O(ND) cost. Population sorting has a complexity of O(NlogN). Aggregating these components, the total time complexity is O(ND+T(ND+NlogN)). In practice, this simplifies to O(TND) because *D* dominates the sorting term. GPS-CC-GME thus preserves the linear scaling profile of the baseline metaheuristic.

## 4. Experimental Results and Analysis

This section assesses the proposed algorithm using a standardized benchmark suite. All algorithms were configured with consistent parameter settings for population size (*N*), dimensionality (*D*), maximum function evaluation count (MaxFEs), and independent runs.

### 4.1. Benchmark Functions Overview

The experiments use the CEC2017 benchmark suite [33], which contains 29 test functions representing diverse landscape topologies: unimodal functions (F1–F3), multimodal functions (F4–F10), hybrid functions (F11–F20), and composition functions (F21–F29). Function F2 was excluded because of well-documented design flaws and numerical instability in the official CEC2017 format, following standard evaluation practice. In all experiments, population size was N=100, dimension D=30, and MaxFEs=10000×D. Each algorithm was executed for 30 independent runs. Details are provided in Table 1.

### 4.2. Parameter Sensitivity Analysis ($P_{cc}$)

**Figure 2:**
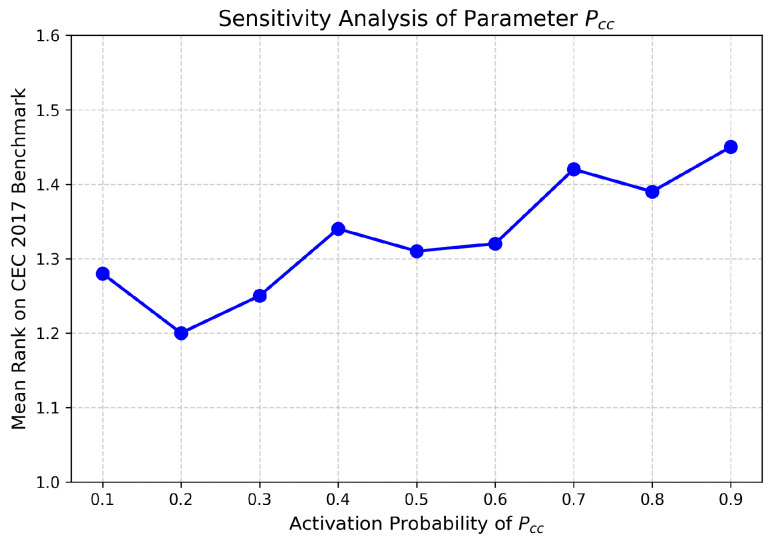
Sensitivity analysis illustrating the mean rank of GPS-CC-GME on the CEC2017 suite under different activation probabilities ($P_{cc}$).

A sensitivity analysis was conducted to evaluate the impact of the CC strategy activation probability, $P_{cc}$. This parameter controls the balance between global exploration (HCS) and local exploitation (VCS). We evaluated $P_{cc}$ values ranging from 0.1 to 0.9. Figure 2 shows the mean rank across all CEC2017 benchmark tests. Optimal performance was observed at $P_{cc}=0.2$; therefore, we configure this as the default parameter.

### 4.3. Ablation Study

An ablation study was conducted on the CEC2017 suite to evaluate the independent contributions of GPS initialization and the CC strategy. Four variants were compared: the original GME, GPS-GME, CC-GME, and GPS-CC-GME. The results across 30 dimensions are summarized in Table 2.

The results in Table 2 show that combining GPS initialization and the CC strategy improves search performance. This combination helps the algorithm escape local optima more effectively than either strategy in isolation, achieving the highest number of “Best” results and the top mean Friedman ranking.

### 4.4. Performance Comparison with Other Algorithms

The performance of GPS-CC-GME is evaluated against GME and eight other metaheuristics: BBO [34], PO [35], ESC [36], DE [24], PSO [21], SMA [37], CPA [38], and MGO [39]. All algorithms were executed under identical experimental conditions.

Because of the computational cost of full runtime profiling across all 28 functions over 30 runs, we profiled execution time exclusively on F1 over 10 runs (Table 3). The results indicate that although GPS-CC-GME yields an incremental runtime cost over canonical GME, its total execution time remains comparable to other variants.

Table 4 details the comparative results, showing the mean fitness (Avg) and standard deviation (Std) over 30 independent runs. The table also provides pairwise statistical tests (+/=/−), indicating whether GPS-CC-GME yielded statistically better (+), equivalent (=), or inferior (−) results. GPS-CC-GME secured the best overall performance with a mean rank of 2.3103, outperforming GME, which achieved a mean rank of 4.3793. In pairwise comparisons, GPS-CC-GME outperformed GME on 23 functions, performed equivalently on 5, and was inferior on 1. Furthermore, GPS-CC-GME exhibited lower standard deviation values across most functions compared to competitors such as SMA and PO. It should be noted that GPS-CC-GME does not achieve the best result on every function. On F3, several algorithms (GME, BBO, PSO, CPA) reach the theoretical optimum, whereas GPS-CC-GME exhibits a minor deviation. On F8, ESC obtains a lower mean fitness value. These cases indicate that the crossover operators do not uniformly benefit all landscape topologies.

The Wilcoxon signed-rank test was conducted (α=0.05) to assess statistical significance; *p*-values are listed in Table 5. GPS-CC-GME shows statistically significant improvement (p<0.05) over GME on unimodal (F1, F3), multimodal (F4–F10), and hybrid functions (F12–F14, F16–F18). For multimodal functions F5 and F6, the low *p*-values ($8.31\times 10^{-10}$ and $4.29\times 10^{-6}$) indicate consistent performance gains. Although mean differences were not statistically significant on some composition functions (e.g., F11, F29), GPS-CC-GME maintained comparative performance levels. Against other competitors, GPS-CC-GME outperformed PO and SMA on all 29 benchmark functions.

**Figure 3:**
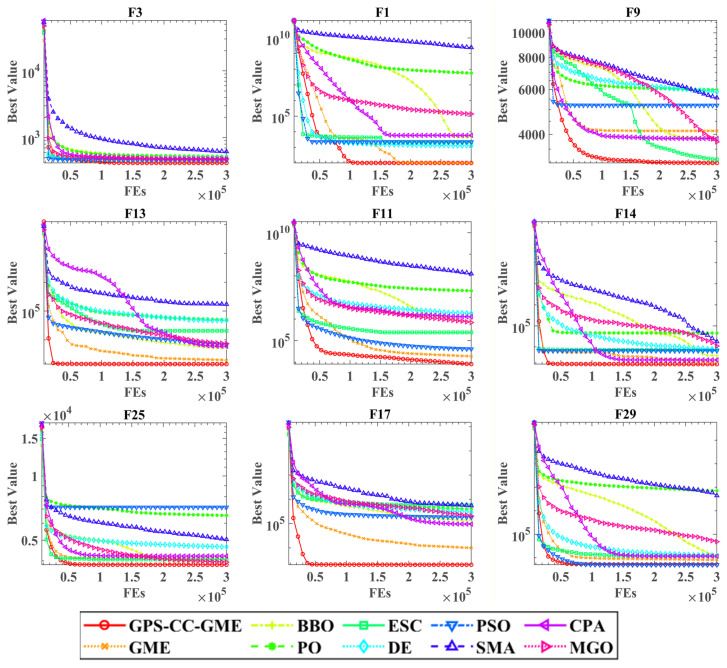
Convergence behavior analysis on nine representative benchmark functions.

Figure 3 illustrates the convergence behavior on representative benchmark functions. The convergence curves show that GPS-CC-GME yields a rapid initial decline in objective fitness, driven by HCS. In later stages, the algorithm maintains a steady improvement trend, preventing search stagnation. This balance between exploration and exploitation allows GPS-CC-GME to converge to higher-quality solutions than the compared algorithms.

**Figure 4:**
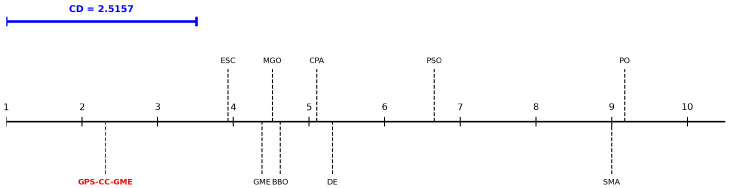
Critical Difference (CD) chart of the Nemenyi test comparing all algorithms on CEC2017 (α=0.05).

A non-parametric Friedman test was conducted across the benchmark functions to quantify statistical significance. The test yielded $\chi^{2}=135.6981$ with a *p*-value of $7.96\times 10^{-25}$, indicating an overall performance variance among the compared algorithms. A Nemenyi post hoc test (coupled with a Bonferroni–Holm step-down correction to control multiple comparison biases) was then applied at α=0.05. The Critical Difference (CD) value computes as CD=2.5157. Based on mean rankings, GPS-CC-GME (2.31) is statistically superior to algorithms with mean ranks exceeding 4.8257, specifically CPA, DE, PSO, SMA, and PO. Although the differences compared to ESC, GME, MGO, and BBO do not exceed the CD threshold, GPS-CC-GME secured the highest overall ranking (Figure 4).

## 5. Application to Production Optimization

Reservoir production optimization identifies operational strategies that maximize hydrocarbon recovery value. The NPV serves as the primary optimization target, formulated as [40]:

(10) $$\mathrm{NPV}\left(x,z\right)=\sum_{t=1}^{n}\frac{\Delta t(Q_{o,t}r_{o}-Q_{w,t}r_{w}-Q_{i,t}r_{i})}{{\left(1+b\right)}^{p_{t}}}$$

where *x* denotes decision variables, *z* denotes reservoir state variables, and $Q_{o,t}$, $Q_{w,t}$, and $Q_{i,t}$ correspond to oil production, water production, and fluid injection rates at time *t*. The terms $r_{o}$, $r_{w}$, and $r_{i}$ represent unit costs, *b* is the annual discount rate, and $p_{t}$ is the cumulative time in years.

### 5.1. Reservoir Model Description

**Figure 5:**
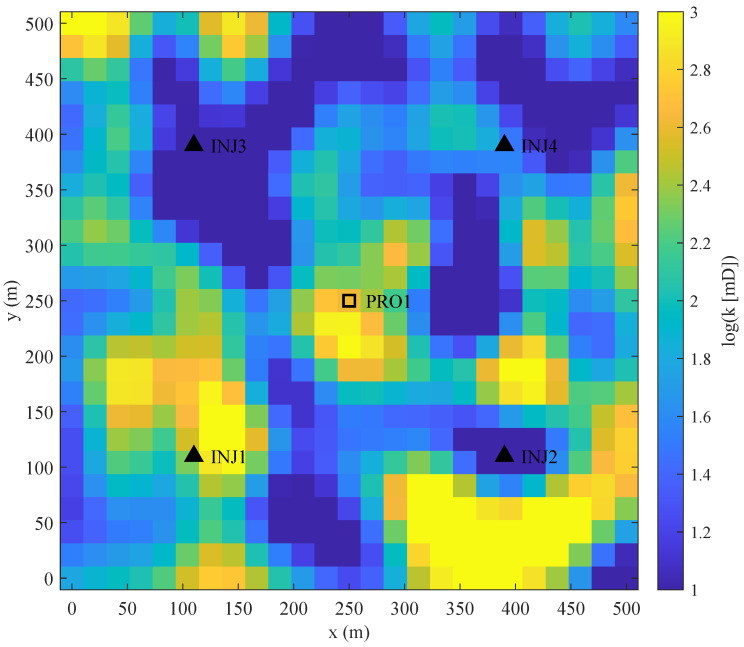
Two-dimensional synthetic reservoir model and well pattern arrangement.

The reservoir model is a synthetic 2D heterogeneous system (Figure 5), used to evaluate optimization performance in complex flow environments. The domain is discretized into a 25×25×1 Cartesian grid with 625 active cells. The well configuration includes five injection wells (INJ1–INJ5) and one production well (PRO1).

The goal is to maximize total NPV over a 1500-day period, divided into 15 control intervals of 100 days. The decision space contains 75 variables, representing water injection rates. Injection rates are bounded between 0 and 1200 STB/DAY. The producer operates at a constant bottom-hole pressure (BHP) of 145 barsa. Economic parameters include an oil price of 85.0 USD/STB and water costs of 3.5 USD/STB. This task involves constraints such as varying injection capacities and BHP limits, operating as a validation case for engineering constraints.

### 5.2. Analysis and Discussion of Experimental Results

GPS-CC-GME is compared against nine metaheuristics on the 75-dimensional reservoir production optimization task. Each method was executed for 30 independent trials, bounded by 100 maximum iterations. Table 6 summarizes the mean, standard deviation (Std), best, and worst NPV.

**Figure 6:**
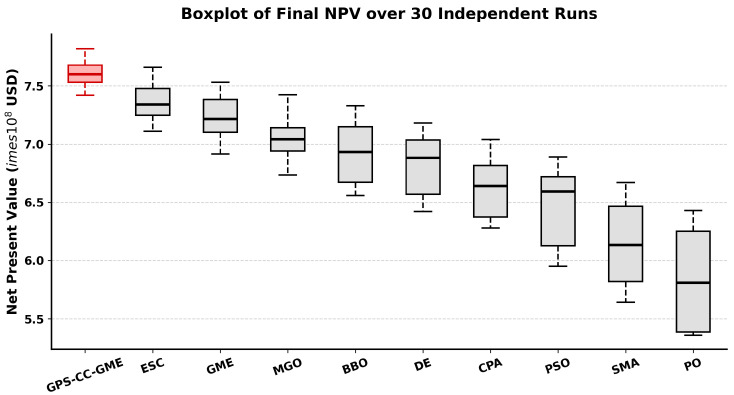
Boxplots of the final NPV distributions obtained by all competing algorithms across 30 independent executions on the reservoir production optimization problem.

The results in Table 6 show that GPS-CC-GME achieved a mean NPV of $7.624\times 10^{8}$ USD, outperforming the compared algorithms. GPS-CC-GME exhibited the lowest standard deviation of $1.285\times 10^{7}$ USD, indicating improved stability relative to GME ($2.147\times 10^{7}$ USD), ESC ($1.893\times 10^{7}$ USD), and CPA ($3.487\times 10^{7}$ USD). Wilcoxon rank-sum test results (p<0.05) indicate that these performance improvements are statistically significant. As shown in the boxplots (Figure 6), GPS-CC-GME maintains the highest median profit and a narrower interquartile range with a higher minimum bound.

**Figure 7:**
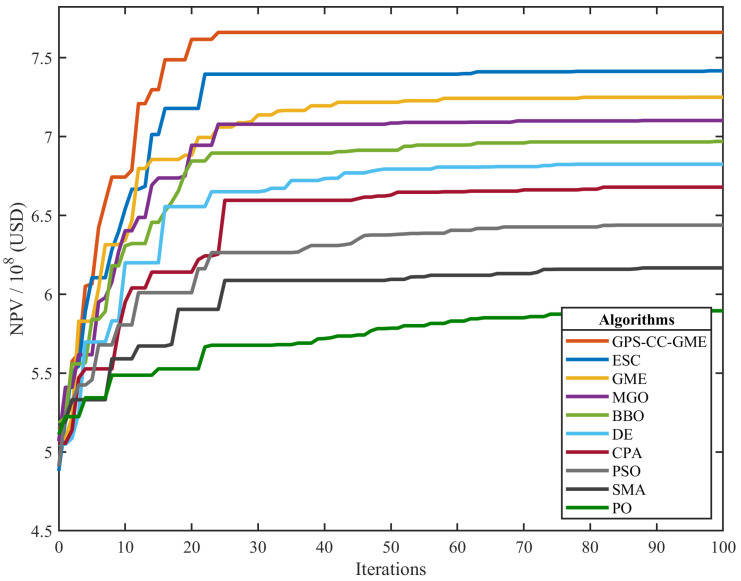
Convergence curve analysis of all algorithms on the production optimization problem.

Figure 7 plots the convergence curves, with the y-axis scaled by $10^{8}$ USD. GPS-CC-GME converges more rapidly than the compared methods, maintaining higher NPV metrics throughout the evaluation. The original GME, MGO, and BBO exhibit strong initial progress but plateau at lower levels. ESC and CPA yield results below GPS-CC-GME, while SMA and PO converge more gradually and stabilize at lower NPV values. The performance gains observed in this engineering scenario can be directly linked to the proposed algorithmic components. The HCS operator facilitates information exchange between distinct candidate solutions, enabling the optimizer to simultaneously explore multiple disconnected geological sub-optima across the heterogeneous permeability field. This global traversal capability is reflected in the rapid initial NPV increase. Complementarily, the VCS operator refines individual injection rate vectors by recombining specific dimensional components, enabling localized fine-tuning of water allocation in high-saturation blocks. This targeted exploitation sustains the optimization trajectory beyond the plateau observed in the baseline GME, ultimately yielding the improved NPV margins reported in Table 6.

## 6. Conclusions

This study proposes GPS-CC-GME, an enhanced algorithmic framework that addresses the structural limitations of the canonical GME by integrating a crisscross (CC) search strategy [41]. By coupling cooperative multipredator hunting dynamics with horizontal and vertical crossover operators, the proposed method establishes a balanced optimization structure. The Good Point Set (GPS) initialization mathematically ensures a uniform starting distribution, while the CC strategy facilitates dimension-wise information exchange, thereby sustaining population diversity and mitigating premature convergence.

Evaluations on the CEC2017 baseline indicate that GPS-CC-GME consistently outperforms the original GME and eight other established metaheuristics, exhibiting the highest overall ranking and stable iteration trajectories across complex landscape topologies. Non-parametric statistical tests [42] validate the significance of these search capability gains. Furthermore, when deployed on a highly-constrained 75-dimensional reservoir production optimization task, the framework yielded a peak output of $7.624\times 10^{8}$ USD in Net Present Value (NPV), establishing a quantified fiscal margin over comparative baselines.

Future research will follow three primary directions. First, we plan to broaden the comparative baseline space and execute evaluations on full 3D fluid flow models, which introduce computational scaling requirements and vertical compartmentalization constraints. In parallel, comprehensive multi-parameter economic sensitivity analysis (e.g., crude price volatility and injection cost fluctuations) will be integrated to validate real-world robustness. Second, while the proposed algorithm efficiently navigated a 75-dimensional engineering space, scaling to ultra-high-dimensional environments (e.g., D≥100 or 500) remains an open challenge; deploying proxy-assisted mechanisms and variable decomposition strategies will be targeted to address the “curse of dimensionality”. Finally, recent studies have demonstrated that hybrid frameworks coupling metaheuristics with machine learning models—such as metaheuristic-trained neural networks and ensemble learners—can yield accurate predictive models in engineering applications [43,44,45]. However, these approaches introduce substantial training overhead. When the raw evaluation cost of the objective function is lower than the parameter-training cost of complex data-driven structures, surrogate models become inefficient. Systematically charting this performance crossover boundary is critical for the practical deployment of future hybrid optimizers.

## Figures and Tables

**Table 1:** CEC2017 benchmark functions.

Function	Function Name	Class	Optimum
F1	Shifted and Rotated Bent Cigar Function	Unimodal	100
F3	Shifted and Rotated Zakharov Function	Unimodal	300
F4	Shifted and Rotated Rosenbrock’s Function	Multimodal	400
F5	Shifted and Rotated Rastrigin’s Function	Multimodal	500
F6	Shifted and Rotated Expanded Scaffer’s F6 Function	Multimodal	600
F7	Shifted and Rotated Lunacek Bi-Rastrigin Function	Multimodal	700
F8	Shifted and Rotated Non-Continuous Rastrigin’s Function	Multimodal	800
F9	Shifted and Rotated Lévy Function	Multimodal	900
F10	Shifted and Rotated Schwefel’s Function	Multimodal	1000
F11	Hybrid Function 1 (N = 3)	Hybrid	1100
F12	Hybrid Function 2 (N = 3)	Hybrid	1200
F13	Hybrid Function 3 (N = 3)	Hybrid	1300
F14	Hybrid Function 4 (N = 4)	Hybrid	1400
F15	Hybrid Function 5 (N = 4)	Hybrid	1500
F16	Hybrid Function 6 (N = 4)	Hybrid	1600
F17	Hybrid Function 7 (N = 5)	Hybrid	1700
F18	Hybrid Function 8 (N = 5)	Hybrid	1800
F19	Hybrid Function 9 (N = 5)	Hybrid	1900
F20	Hybrid Function 10 (N = 6)	Hybrid	2000
F21	Composition Function 1 (N = 3)	Composition	2100
F22	Composition Function 2 (N = 3)	Composition	2200
F23	Composition Function 3 (N = 4)	Composition	2300
F24	Composition Function 4 (N = 4)	Composition	2400
F25	Composition Function 5 (N = 5)	Composition	2500
F26	Composition Function 6 (N = 5)	Composition	2600
F27	Composition Function 7 (N = 6)	Composition	2700
F28	Composition Function 8 (N = 6)	Composition	2800
F29	Composition Function 9 (N = 3)	Composition	2900
F30	Composition Function 10 (N = 3)	Composition	3000

**Table 2:** Statistical summary of GPS-CC-GME and its variants on CEC 2017 (30D). “Best” denotes the number of functions where the variant achieved the best result. “Mean Rank” is the average Friedman ranking. “+/≈/−” summarizes Wilcoxon test outcomes (α=0.05). Best results are highlighted.

Algorithm	Best	Mean Rank	+/≈/−
GPS-CC-GME (Proposed)	19	1.24	—
GPS-GME	4	2.38	18/9/2
CC-GME	4	2.62	20/7/2
GME (Original)	2	3.76	25/4/0

**Table 3:** Execution time (s) of competing algorithms for F1 over 10 independent test operations.

Algorithm	Runtime over 10 Runs (s)
GPS-CC-GME (Ours)	21.5
GME	18.4
BBO	25.2
PO	38.9
ESC	27.6
DE	15.5
PSO	16.2
SMA	34.5
CPA	31.4
MGO	20.8

**Table 4:** Results of the GPS-CC-GME and Other Algorithms on CEC2017 Benchmark Functions.

	**F1**		**F3**		**F4**	
**Algo.**	**Avg**	**Std**	**Avg**	**Std**	**Avg**	**Std**
GPS-CC-GME	$1.0000\times 10^{2}$	$3.0887\times 10^{-14}$	$3.0109\times 10^{2}$	$3.4573\times 10^{0}$	$4.1749\times 10^{2}$	$2.7218\times 10^{1}$
GME	$1.0014\times 10^{2}$	$6.0510\times 10^{-1}$	$3.0000\times 10^{2}$	$6.2482\times 10^{-6}$	$4.4202\times 10^{2}$	$3.7374\times 10^{1}$
BBO	$4.5276\times 10^{3}$	$5.9550\times 10^{3}$	$3.0000\times 10^{2}$	$2.2107\times 10^{-3}$	$4.7570\times 10^{2}$	$3.2674\times 10^{1}$
PO	$5.6758\times 10^{7}$	$1.3192\times 10^{8}$	$5.6837\times 10^{3}$	$3.0178\times 10^{3}$	$5.2518\times 10^{2}$	$3.7100\times 10^{1}$
ESC	$2.1950\times 10^{3}$	$1.8162\times 10^{3}$	$6.0449\times 10^{2}$	$4.8201\times 10^{2}$	$4.6512\times 10^{2}$	$2.9665\times 10^{1}$
DE	$1.2710\times 10^{3}$	$2.3005\times 10^{3}$	$1.8425\times 10^{4}$	$3.8520\times 10^{3}$	$4.9234\times 10^{2}$	$1.1507\times 10^{1}$
PSO	$2.1939\times 10^{3}$	$2.1750\times 10^{3}$	$3.0000\times 10^{2}$	$3.7208\times 10^{-3}$	$4.6126\times 10^{2}$	$2.8734\times 10^{1}$
SMA	$2.4788\times 10^{9}$	$1.0156\times 10^{9}$	$4.1207\times 10^{4}$	$9.3867\times 10^{3}$	$6.1911\times 10^{2}$	$5.6986\times 10^{1}$
CPA	$5.8308\times 10^{3}$	$5.7763\times 10^{3}$	$3.0000\times 10^{2}$	$1.5847\times 10^{-7}$	$4.7403\times 10^{2}$	$3.5175\times 10^{1}$
MGO	$1.3694\times 10^{5}$	$2.0151\times 10^{5}$	$6.0260\times 10^{3}$	$2.4768\times 10^{3}$	$4.8043\times 10^{2}$	$2.0862\times 10^{1}$
	**F5**		**F6**		**F7**	
**Algo.**	**Avg**	**Std**	**Avg**	**Std**	**Avg**	**Std**
GPS-CC-GME	$5.6024\times 10^{2}$	$1.1907\times 10^{1}$	$6.0000\times 10^{2}$	$5.9817\times 10^{-4}$	$7.8926\times 10^{2}$	$1.2761\times 10^{1}$
GME	$6.1390\times 10^{2}$	$2.9194\times 10^{1}$	$6.0000\times 10^{2}$	$6.5403\times 10^{-3}$	$8.5931\times 10^{2}$	$3.3688\times 10^{1}$
BBO	$5.7769\times 10^{2}$	$1.7496\times 10^{1}$	$6.0062\times 10^{2}$	$1.2610\times 10^{0}$	$8.2449\times 10^{2}$	$2.0467\times 10^{1}$
PO	$7.2579\times 10^{2}$	$4.3381\times 10^{1}$	$6.5532\times 10^{2}$	$1.1118\times 10^{1}$	$1.1550\times 10^{3}$	$8.7184\times 10^{1}$
ESC	$5.2776\times 10^{2}$	$1.0016\times 10^{1}$	$6.0016\times 10^{2}$	$2.1313\times 10^{-1}$	$7.6452\times 10^{2}$	$1.4030\times 10^{1}$
DE	$6.0838\times 10^{2}$	$8.9967\times 10^{0}$	$6.0000\times 10^{2}$	$0.0000\times 10^{0}$	$8.4137\times 10^{2}$	$9.2799\times 10^{0}$
PSO	$7.0794\times 10^{2}$	$3.0352\times 10^{1}$	$6.4683\times 10^{2}$	$7.5110\times 10^{0}$	$1.0190\times 10^{3}$	$5.9091\times 10^{1}$
SMA	$7.2163\times 10^{2}$	$3.9974\times 10^{1}$	$6.4288\times 10^{2}$	$9.6476\times 10^{0}$	$1.0783\times 10^{3}$	$5.4670\times 10^{1}$
CPA	$6.2848\times 10^{2}$	$3.0089\times 10^{1}$	$6.0000\times 10^{2}$	$4.9821\times 10^{-4}$	$8.4512\times 10^{2}$	$3.0295\times 10^{1}$
MGO	$5.4922\times 10^{2}$	$9.2542\times 10^{0}$	$6.0000\times 10^{2}$	$9.2582\times 10^{-5}$	$7.8203\times 10^{2}$	$1.2318\times 10^{1}$
	**F8**		**F9**		**F10**	
**Algo.**	**Avg**	**Std**	**Avg**	**Std**	**Avg**	**Std**
GPS-CC-GME	$8.6346\times 10^{2}$	$1.1520\times 10^{1}$	$9.5960\times 10^{2}$	$5.4777\times 10^{1}$	$3.0873\times 10^{3}$	$2.9180\times 10^{2}$
GME	$9.0928\times 10^{2}$	$2.4022\times 10^{1}$	$1.4491\times 10^{3}$	$5.9512\times 10^{2}$	$4.1249\times 10^{3}$	$4.8072\times 10^{2}$
BBO	$8.6918\times 10^{2}$	$2.0795\times 10^{1}$	$1.1094\times 10^{3}$	$3.2944\times 10^{2}$	$3.8754\times 10^{3}$	$5.2925\times 10^{2}$
PO	$9.6239\times 10^{2}$	$2.6889\times 10^{1}$	$5.0473\times 10^{3}$	$9.2649\times 10^{2}$	$5.9819\times 10^{3}$	$7.7139\times 10^{2}$
ESC	$8.3171\times 10^{2}$	$9.3032\times 10^{0}$	$9.2453\times 10^{2}$	$2.5983\times 10^{1}$	$3.1852\times 10^{3}$	$8.4556\times 10^{2}$
DE	$9.0994\times 10^{2}$	$8.3325\times 10^{0}$	$9.0000\times 10^{2}$	$1.0125\times 10^{-13}$	$5.8693\times 10^{3}$	$2.9131\times 10^{2}$
PSO	$9.3591\times 10^{2}$	$3.2504\times 10^{1}$	$4.3363\times 10^{3}$	$7.2274\times 10^{2}$	$5.1947\times 10^{3}$	$7.4234\times 10^{2}$
SMA	$9.6920\times 10^{2}$	$2.7057\times 10^{1}$	$5.5305\times 10^{3}$	$1.0614\times 10^{3}$	$5.5638\times 10^{3}$	$7.3024\times 10^{2}$
CPA	$9.0590\times 10^{2}$	$1.5208\times 10^{1}$	$2.3029\times 10^{3}$	$5.6061\times 10^{2}$	$3.8506\times 10^{3}$	$5.3070\times 10^{2}$
MGO	$8.5180\times 10^{2}$	$1.0949\times 10^{1}$	$9.0440\times 10^{2}$	$4.8883\times 10^{0}$	$3.7346\times 10^{3}$	$4.1472\times 10^{2}$
	**F11**		**F12**		**F13**	
**Algo.**	**Avg**	**Std**	**Avg**	**Std**	**Avg**	**Std**
GPS-CC-GME	$1.1291\times 10^{3}$	$1.7191\times 10^{1}$	$8.1070\times 10^{3}$	$1.0997\times 10^{4}$	$1.3411\times 10^{3}$	$1.9194\times 10^{1}$
GME	$1.1773\times 10^{3}$	$2.9521\times 10^{1}$	$1.8677\times 10^{4}$	$1.1734\times 10^{4}$	$6.0196\times 10^{3}$	$7.4261\times 10^{3}$
BBO	$1.2237\times 10^{3}$	$4.4543\times 10^{1}$	$7.5440\times 10^{5}$	$4.8667\times 10^{5}$	$1.8211\times 10^{4}$	$8.5502\times 10^{3}$
PO	$1.3047\times 10^{3}$	$8.2183\times 10^{1}$	$2.0357\times 10^{7}$	$1.5238\times 10^{7}$	$1.0773\times 10^{5}$	$9.8054\times 10^{4}$
ESC	$1.1672\times 10^{3}$	$3.1552\times 10^{1}$	$2.4048\times 10^{5}$	$2.0445\times 10^{5}$	$1.5767\times 10^{4}$	$1.2512\times 10^{4}$
DE	$1.1508\times 10^{3}$	$2.7424\times 10^{1}$	$1.8308\times 10^{6}$	$7.9613\times 10^{5}$	$3.0804\times 10^{4}$	$1.8396\times 10^{4}$
PSO	$1.2104\times 10^{3}$	$3.1540\times 10^{1}$	$4.0494\times 10^{4}$	$2.8624\times 10^{4}$	$2.1407\times 10^{4}$	$2.1846\times 10^{4}$
SMA	$1.5707\times 10^{3}$	$1.3253\times 10^{2}$	$1.2550\times 10^{8}$	$5.6333\times 10^{7}$	$2.8084\times 10^{6}$	$5.2012\times 10^{6}$
CPA	$1.1667\times 10^{3}$	$3.1082\times 10^{1}$	$1.2781\times 10^{6}$	$9.4737\times 10^{5}$	$3.5471\times 10^{3}$	$2.9136\times 10^{3}$
MGO	$1.1863\times 10^{3}$	$2.3620\times 10^{1}$	$6.8652\times 10^{5}$	$5.4344\times 10^{5}$	$2.3992\times 10^{4}$	$1.6094\times 10^{4}$
	**F14**		**F15**		**F16**	
**Algo.**	**Avg**	**Std**	**Avg**	**Std**	**Avg**	**Std**
GPS-CC-GME	$1.4314\times 10^{3}$	$1.3234\times 10^{1}$	$1.5226\times 10^{3}$	$1.8664\times 10^{1}$	$2.1752\times 10^{3}$	$1.7047\times 10^{2}$
GME	$1.9178\times 10^{3}$	$1.0803\times 10^{3}$	$1.9480\times 10^{3}$	$8.7025\times 10^{2}$	$2.4912\times 10^{3}$	$3.2872\times 10^{2}$
BBO	$5.2329\times 10^{3}$	$3.2085\times 10^{3}$	$3.8221\times 10^{3}$	$2.7457\times 10^{3}$	$2.3589\times 10^{3}$	$2.0882\times 10^{2}$
PO	$5.0807\times 10^{4}$	$7.6091\times 10^{4}$	$4.5569\times 10^{4}$	$3.3811\times 10^{4}$	$3.1708\times 10^{3}$	$3.6796\times 10^{2}$
ESC	$2.0514\times 10^{4}$	$2.0392\times 10^{4}$	$7.2804\times 10^{3}$	$6.0815\times 10^{3}$	$1.9779\times 10^{3}$	$2.3327\times 10^{2}$
DE	$4.6875\times 10^{4}$	$2.8277\times 10^{4}$	$7.8336\times 10^{3}$	$4.0390\times 10^{3}$	$2.0505\times 10^{3}$	$1.4939\times 10^{2}$
PSO	$6.7858\times 10^{3}$	$3.2195\times 10^{3}$	$6.5495\times 10^{3}$	$5.4266\times 10^{3}$	$2.9114\times 10^{3}$	$3.2463\times 10^{2}$
SMA	$1.7292\times 10^{5}$	$9.2052\times 10^{4}$	$1.7907\times 10^{4}$	$8.9464\times 10^{3}$	$2.7680\times 10^{3}$	$3.2345\times 10^{2}$
CPA	$5.9852\times 10^{3}$	$4.6350\times 10^{3}$	$2.4107\times 10^{3}$	$1.1485\times 10^{3}$	$2.7707\times 10^{3}$	$2.8421\times 10^{2}$
MGO	$7.3927\times 10^{3}$	$4.7318\times 10^{3}$	$1.1061\times 10^{4}$	$6.1761\times 10^{3}$	$2.1397\times 10^{3}$	$1.1209\times 10^{2}$
	**F17**		**F18**		**F19**	
**Algo.**	**Avg**	**Std**	**Avg**	**Std**	**Avg**	**Std**
GPS-CC-GME	$1.8753\times 10^{3}$	$1.0815\times 10^{2}$	$1.9753\times 10^{3}$	$6.1872\times 10^{2}$	$1.9137\times 10^{3}$	$4.3386\times 10^{0}$
GME	$2.0583\times 10^{3}$	$2.3066\times 10^{2}$	$9.9460\times 10^{3}$	$7.9896\times 10^{3}$	$1.9872\times 10^{3}$	$7.6119\times 10^{1}$
BBO	$1.9179\times 10^{3}$	$1.2024\times 10^{2}$	$1.3795\times 10^{5}$	$9.4970\times 10^{4}$	$7.3249\times 10^{3}$	$3.8358\times 10^{3}$
PO	$2.2284\times 10^{3}$	$1.8550\times 10^{2}$	$4.0018\times 10^{5}$	$2.6951\times 10^{5}$	$8.3723\times 10^{5}$	$6.8699\times 10^{5}$
ESC	$1.8401\times 10^{3}$	$1.0819\times 10^{2}$	$5.8384\times 10^{5}$	$8.1296\times 10^{5}$	$8.4748\times 10^{3}$	$8.1136\times 10^{3}$
DE	$1.8443\times 10^{3}$	$5.3833\times 10^{1}$	$2.9503\times 10^{5}$	$1.6702\times 10^{5}$	$7.9575\times 10^{3}$	$4.8821\times 10^{3}$
PSO	$2.4473\times 10^{3}$	$2.5402\times 10^{2}$	$1.7825\times 10^{5}$	$1.5256\times 10^{5}$	$7.8803\times 10^{3}$	$7.4373\times 10^{3}$
SMA	$2.3209\times 10^{3}$	$1.8236\times 10^{2}$	$5.4812\times 10^{5}$	$4.3251\times 10^{5}$	$3.6456\times 10^{5}$	$3.6729\times 10^{5}$
CPA	$2.1930\times 10^{3}$	$2.2556\times 10^{2}$	$9.4859\times 10^{4}$	$4.6061\times 10^{4}$	$5.0947\times 10^{3}$	$2.0439\times 10^{3}$
MGO	$1.8629\times 10^{3}$	$5.3605\times 10^{1}$	$2.0458\times 10^{5}$	$9.6097\times 10^{4}$	$8.8420\times 10^{3}$	$6.4589\times 10^{3}$
	**F20**		**F21**		**F22**	
**Algo.**	**Avg**	**Std**	**Avg**	**Std**	**Avg**	**Std**
GPS-CC-GME	$2.1726\times 10^{3}$	$9.0251\times 10^{1}$	$2.3736\times 10^{3}$	$1.2840\times 10^{1}$	$2.8680\times 10^{3}$	$1.0651\times 10^{3}$
GME	$2.3309\times 10^{3}$	$1.3286\times 10^{2}$	$2.4044\times 10^{3}$	$2.6937\times 10^{1}$	$2.3013\times 10^{3}$	$2.0296\times 10^{0}$
BBO	$2.2702\times 10^{3}$	$8.2509\times 10^{1}$	$2.3700\times 10^{3}$	$1.8280\times 10^{1}$	$2.7955\times 10^{3}$	$1.1318\times 10^{3}$
PO	$2.4900\times 10^{3}$	$1.4810\times 10^{2}$	$2.5037\times 10^{3}$	$3.8785\times 10^{1}$	$3.3567\times 10^{3}$	$1.8348\times 10^{3}$
ESC	$2.1841\times 10^{3}$	$1.5569\times 10^{2}$	$2.3286\times 10^{3}$	$7.2835\times 10^{0}$	$2.9867\times 10^{3}$	$1.2417\times 10^{3}$
DE	$2.1568\times 10^{3}$	$6.6707\times 10^{1}$	$2.4077\times 10^{3}$	$9.5386\times 10^{0}$	$4.2100\times 10^{3}$	$2.0009\times 10^{3}$
PSO	$2.6746\times 10^{3}$	$1.8364\times 10^{2}$	$2.4745\times 10^{3}$	$4.4151\times 10^{1}$	$5.1284\times 10^{3}$	$2.2694\times 10^{3}$
SMA	$2.4637\times 10^{3}$	$1.2846\times 10^{2}$	$2.4759\times 10^{3}$	$2.8831\times 10^{1}$	$3.5831\times 10^{3}$	$1.8436\times 10^{3}$
CPA	$2.4349\times 10^{3}$	$1.6734\times 10^{2}$	$2.3716\times 10^{3}$	$9.0432\times 10^{1}$	$3.0016\times 10^{3}$	$1.4639\times 10^{3}$
MGO	$2.1869\times 10^{3}$	$6.6903\times 10^{1}$	$2.3542\times 10^{3}$	$9.2461\times 10^{0}$	$2.9561\times 10^{3}$	$1.2002\times 10^{3}$
	**F23**		**F24**		**F25**	
**Algo.**	**Avg**	**Std**	**Avg**	**Std**	**Avg**	**Std**
GPS-CC-GME	$2.7171\times 10^{3}$	$1.6000\times 10^{1}$	$2.9463\times 10^{3}$	$3.0209\times 10^{1}$	$2.8859\times 10^{3}$	$1.6300\times 10^{0}$
GME	$2.7525\times 10^{3}$	$2.5106\times 10^{1}$	$2.9492\times 10^{3}$	$3.6914\times 10^{1}$	$2.8997\times 10^{3}$	$1.8733\times 10^{1}$
BBO	$2.7262\times 10^{3}$	$2.1170\times 10^{1}$	$2.8844\times 10^{3}$	$1.7058\times 10^{1}$	$2.8881\times 10^{3}$	$3.1585\times 10^{0}$
PO	$2.9669\times 10^{3}$	$6.6860\times 10^{1}$	$3.0962\times 10^{3}$	$5.4651\times 10^{1}$	$2.9337\times 10^{3}$	$2.9780\times 10^{1}$
ESC	$2.6865\times 10^{3}$	$1.1343\times 10^{1}$	$2.8533\times 10^{3}$	$1.0856\times 10^{1}$	$2.8988\times 10^{3}$	$1.6195\times 10^{1}$
DE	$2.7576\times 10^{3}$	$8.7344\times 10^{0}$	$2.9547\times 10^{3}$	$1.1443\times 10^{1}$	$2.8874\times 10^{3}$	$6.8520\times 10^{-1}$
PSO	$3.3165\times 10^{3}$	$1.5437\times 10^{2}$	$3.3353\times 10^{3}$	$9.2012\times 10^{1}$	$2.8834\times 10^{3}$	$1.2906\times 10^{1}$
SMA	$2.8604\times 10^{3}$	$3.0504\times 10^{1}$	$3.0148\times 10^{3}$	$3.7991\times 10^{1}$	$2.9937\times 10^{3}$	$3.5447\times 10^{1}$
CPA	$2.7520\times 10^{3}$	$2.4638\times 10^{1}$	$3.0484\times 10^{3}$	$7.6144\times 10^{1}$	$2.8928\times 10^{3}$	$1.0421\times 10^{1}$
MGO	$2.7087\times 10^{3}$	$1.2027\times 10^{1}$	$2.8731\times 10^{3}$	$4.6958\times 10^{1}$	$2.8875\times 10^{3}$	$1.8097\times 10^{0}$
	**F26**		**F27**		**F28**	
**Algo.**	**Avg**	**Std**	**Avg**	**Std**	**Avg**	**Std**
GPS-CC-GME	$3.8355\times 10^{3}$	$7.6110\times 10^{2}$	$3.2106\times 10^{3}$	$1.5840\times 10^{1}$	$3.1459\times 10^{3}$	$6.2742\times 10^{1}$
GME	$4.1348\times 10^{3}$	$1.2881\times 10^{3}$	$3.2335\times 10^{3}$	$1.5828\times 10^{1}$	$3.1480\times 10^{3}$	$5.6610\times 10^{1}$
BBO	$3.9737\times 10^{3}$	$8.9985\times 10^{2}$	$3.2162\times 10^{3}$	$1.1816\times 10^{1}$	$3.1674\times 10^{3}$	$5.4165\times 10^{1}$
PO	$6.5343\times 10^{3}$	$1.7140\times 10^{3}$	$3.3193\times 10^{3}$	$7.0766\times 10^{1}$	$3.2961\times 10^{3}$	$3.2038\times 10^{1}$
ESC	$4.0667\times 10^{3}$	$1.9471\times 10^{2}$	$3.2295\times 10^{3}$	$1.1424\times 10^{1}$	$3.1832\times 10^{3}$	$4.9404\times 10^{1}$
DE	$4.6563\times 10^{3}$	$1.0238\times 10^{2}$	$3.2040\times 10^{3}$	$3.2385\times 10^{0}$	$3.1902\times 10^{3}$	$4.7969\times 10^{1}$
PSO	$7.1306\times 10^{3}$	$2.0541\times 10^{3}$	$3.2934\times 10^{3}$	$2.9299\times 10^{2}$	$3.1635\times 10^{3}$	$6.4101\times 10^{1}$
SMA	$5.0415\times 10^{3}$	$7.0363\times 10^{2}$	$3.2562\times 10^{3}$	$2.8144\times 10^{1}$	$3.3975\times 10^{3}$	$3.8534\times 10^{1}$
CPA	$4.2153\times 10^{3}$	$1.4580\times 10^{3}$	$3.2384\times 10^{3}$	$1.4883\times 10^{1}$	$3.1641\times 10^{3}$	$4.3358\times 10^{1}$
MGO	$3.9450\times 10^{3}$	$4.1285\times 10^{2}$	$3.2078\times 10^{3}$	$5.3565\times 10^{0}$	$3.2230\times 10^{3}$	$1.4515\times 10^{1}$
	**F29**		**F30**			
**Algo.**	**Avg**	**Std**	**Avg**	**Std**		
GPS-CC-GME	$3.4425\times 10^{3}$	$8.8689\times 10^{1}$	$5.1591\times 10^{3}$	$1.8941\times 10^{2}$		
GME	$3.7096\times 10^{3}$	$1.8084\times 10^{2}$	$8.1053\times 10^{3}$	$2.0932\times 10^{3}$		
BBO	$3.5551\times 10^{3}$	$1.2355\times 10^{2}$	$1.2771\times 10^{4}$	$3.4435\times 10^{3}$		
PO	$4.5324\times 10^{3}$	$3.5620\times 10^{2}$	$7.0501\times 10^{6}$	$4.2784\times 10^{6}$		
ESC	$3.5433\times 10^{3}$	$1.2552\times 10^{2}$	$1.2075\times 10^{4}$	$3.2705\times 10^{3}$		
DE	$3.5022\times 10^{3}$	$6.5315\times 10^{1}$	$1.2134\times 10^{4}$	$3.0569\times 10^{3}$		
PSO	$3.9449\times 10^{3}$	$2.9592\times 10^{2}$	$5.0236\times 10^{3}$	$2.6629\times 10^{3}$		
SMA	$3.9892\times 10^{3}$	$2.5043\times 10^{2}$	$4.4073\times 10^{6}$	$2.7186\times 10^{6}$		
CPA	$3.6686\times 10^{3}$	$2.1781\times 10^{2}$	$1.1678\times 10^{4}$	$4.1006\times 10^{3}$		
MGO	$3.5930\times 10^{3}$	$7.9519\times 10^{1}$	$4.8393\times 10^{4}$	$3.2726\times 10^{4}$		
**Overall Rank**						
**Algo.**	**RANK**	**+/=/**−	**AVG**			
GPS-CC-GME	1	∼	2.3103			
GME	3	23/5/1	4.3793			
BBO	5	19/8/2	4.6207			
PO	10	29/0/0	9.1724			
ESC	2	16/5/8	3.931			
DE	7	22/3/4	5.3103			
PSO	8	24/3/2	6.6552			
SMA	9	29/0/0	9.0			
CPA	6	24/3/2	5.1034			
MGO	4	15/6/8	4.5172			

*Note*: The bold numbers indicate the best results among all compared algorithms.

**Table 5:** The *p*-values of the GPS-CC-GME versus other algorithms on CEC2017.

**Fun**	**GPS-CC-GME**	**GME**	**BBO**	**PO**	**ESC**
F1	/	$1.20\times 10^{-1}$	$1.73\times 10^{-6}$	$1.73\times 10^{-6}$	$1.73\times 10^{-6}$
F3	/	$1.73\times 10^{-6}$	$1.73\times 10^{-6}$	$1.73\times 10^{-6}$	$4.73\times 10^{-6}$
F4	/	$3.09\times 10^{-1}$	$1.73\times 10^{-6}$	$1.92\times 10^{-10}$	$1.92\times 10^{-10}$
F5	/	$2.35\times 10^{-6}$	$1.73\times 10^{-6}$	$1.73\times 10^{-6}$	$1.73\times 10^{-6}$
F6	/	$8.31\times 10^{-10}$	$1.73\times 10^{-6}$	$1.73\times 10^{-6}$	$1.73\times 10^{-6}$
F7	/	$4.29\times 10^{-6}$	$1.73\times 10^{-6}$	$1.73\times 10^{-6}$	$1.73\times 10^{-6}$
F8	/	$1.49\times 10^{-5}$	$1.73\times 10^{-10}$	$2.35\times 10^{-10}$	$1.73\times 10^{-6}$
F9	/	$2.60\times 10^{-6}$	$1.73\times 10^{-10}$	$1.73\times 10^{-6}$	$1.73\times 10^{-6}$
F10	/	$5.22\times 10^{-6}$	$1.73\times 10^{-10}$	$2.56\times 10^{-10}$	$1.92\times 10^{-10}$
F11	/	$3.87\times 10^{-2}$	$1.73\times 10^{-10}$	$1.73\times 10^{-10}$	$1.73\times 10^{-10}$
F12	/	$7.81\times 10^{-1}$	$1.73\times 10^{-10}$	$1.73\times 10^{-10}$	$1.73\times 10^{-10}$
F13	/	$3.60\times 10^{-1}$	$1.73\times 10^{-10}$	$3.88\times 10^{-6}$	$3.52\times 10^{-6}$
F14	/	$4.90\times 10^{-4}$	$1.73\times 10^{-10}$	$1.73\times 10^{-10}$	$1.73\times 10^{-10}$
F15	/	$1.71\times 10^{-3}$	$1.73\times 10^{-10}$	$1.73\times 10^{-10}$	$2.35\times 10^{-10}$
F16	/	$7.34\times 10^{-1}$	$1.92\times 10^{-10}$	$2.11\times 10^{-3}$	$1.73\times 10^{-10}$
F17	/	$6.84\times 10^{-3}$	$1.73\times 10^{-10}$	$1.64\times 10^{-5}$	$1.73\times 10^{-10}$
F18	/	$2.56\times 10^{-2}$	$3.18\times 10^{-6}$	$4.20\times 10^{-4}$	$7.71\times 10^{-4}$
F19	/	$7.27\times 10^{-3}$	$1.73\times 10^{-10}$	$1.73\times 10^{-6}$	$2.60\times 10^{-6}$
F20	/	$1.47\times 10^{-1}$	$1.73\times 10^{-10}$	$1.48\times 10^{-4}$	$1.92\times 10^{-6}$
F21	/	$2.18\times 10^{-2}$	$1.73\times 10^{-10}$	$4.45\times 10^{-5}$	$1.73\times 10^{-6}$
F22	/	$4.95\times 10^{-2}$	$4.73\times 10^{-10}$	$3.72\times 10^{-5}$	$6.34\times 10^{-10}$
F23	/	$1.57\times 10^{-2}$	$1.73\times 10^{-10}$	$2.60\times 10^{-5}$	$1.92\times 10^{-10}$
F24	/	$5.67\times 10^{-3}$	$1.73\times 10^{-10}$	$5.22\times 10^{-10}$	$1.73\times 10^{-6}$
F25	/	$8.59\times 10^{-2}$	$1.73\times 10^{-10}$	$1.73\times 10^{-10}$	$1.92\times 10^{-6}$
F26	/	$6.29\times 10^{-1}$	$1.92\times 10^{-10}$	$1.24\times 10^{-5}$	$1.73\times 10^{-6}$
F27	/	$1.31\times 10^{-1}$	$1.73\times 10^{-10}$	$1.92\times 10^{-10}$	$1.73\times 10^{-6}$
F28	/	$3.00\times 10^{-2}$	$1.73\times 10^{-10}$	$1.73\times 10^{-6}$	$1.73\times 10^{-6}$
F29	/	$9.59\times 10^{-1}$	$1.73\times 10^{-10}$	$2.22\times 10^{-4}$	$2.35\times 10^{-6}$
F30	/	$1.38\times 10^{-3}$	$1.73\times 10^{-10}$	$1.73\times 10^{-10}$	$1.80\times 10^{-5}$
**Fun**	**DE**	**PSO**	**SMA**	**CPA**	**MGO**
F1	$1.73\times 10^{-6}$	$4.73\times 10^{-6}$	$1.73\times 10^{-6}$	$5.22\times 10^{-6}$	$1.73\times 10^{-6}$
F3	$1.73\times 10^{-6}$	$1.73\times 10^{-6}$	$1.73\times 10^{-6}$	$1.73\times 10^{-6}$	$1.73\times 10^{-6}$
F4	$1.73\times 10^{-6}$	$3.18\times 10^{-6}$	$1.73\times 10^{-6}$	$1.48\times 10^{-2}$	$1.73\times 10^{-6}$
F5	$1.73\times 10^{-6}$	$1.73\times 10^{-6}$	$1.73\times 10^{-6}$	$1.73\times 10^{-6}$	$1.73\times 10^{-6}$
F6	$1.73\times 10^{-6}$	$1.73\times 10^{-6}$	$1.73\times 10^{-6}$	$1.73\times 10^{-6}$	$1.73\times 10^{-6}$
F7	$1.73\times 10^{-6}$	$1.73\times 10^{-6}$	$1.73\times 10^{-6}$	$1.73\times 10^{-6}$	$1.73\times 10^{-6}$
F8	$1.73\times 10^{-6}$	$1.73\times 10^{-6}$	$1.73\times 10^{-6}$	$1.73\times 10^{-6}$	$1.73\times 10^{-6}$
F9	$1.73\times 10^{-6}$	$1.73\times 10^{-6}$	$1.73\times 10^{-6}$	$1.73\times 10^{-6}$	$1.73\times 10^{-6}$
F10	$1.73\times 10^{-6}$	$4.29\times 10^{-6}$	$1.92\times 10^{-10}$	$1.73\times 10^{-10}$	$1.73\times 10^{-10}$
F11	$1.73\times 10^{-6}$	$1.40\times 10^{-2}$	$1.73\times 10^{-10}$	$1.73\times 10^{-10}$	$1.73\times 10^{-10}$
F12	$1.73\times 10^{-6}$	$1.73\times 10^{-10}$	$1.73\times 10^{-10}$	$7.69\times 10^{-6}$	$1.73\times 10^{-6}$
F13	$1.73\times 10^{-6}$	$9.37\times 10^{-2}$	$1.73\times 10^{-10}$	$1.73\times 10^{-6}$	$1.73\times 10^{-6}$
F14	$1.73\times 10^{-6}$	$6.00\times 10^{-1}$	$1.73\times 10^{-10}$	$6.58\times 10^{-1}$	$1.73\times 10^{-6}$
F15	$1.73\times 10^{-6}$	$5.32\times 10^{-3}$	$1.11\times 10^{-3}$	$1.73\times 10^{-10}$	$1.73\times 10^{-6}$
F16	$1.73\times 10^{-6}$	$2.60\times 10^{-6}$	$1.92\times 10^{-10}$	$1.73\times 10^{-10}$	$1.73\times 10^{-6}$
F17	$1.73\times 10^{-6}$	$1.92\times 10^{-10}$	$2.35\times 10^{-6}$	$1.73\times 10^{-10}$	$1.73\times 10^{-6}$
F18	$1.73\times 10^{-6}$	$1.96\times 10^{-2}$	$2.22\times 10^{-4}$	$4.17\times 10^{-1}$	$1.73\times 10^{-6}$
F19	$1.73\times 10^{-6}$	$5.04\times 10^{-1}$	$3.52\times 10^{-10}$	$1.73\times 10^{-6}$	$1.73\times 10^{-6}$
F20	$1.73\times 10^{-6}$	$1.73\times 10^{-10}$	$2.35\times 10^{-6}$	$1.73\times 10^{-10}$	$1.73\times 10^{-6}$
F21	$1.73\times 10^{-6}$	$1.73\times 10^{-10}$	$1.73\times 10^{-10}$	$1.73\times 10^{-10}$	$1.73\times 10^{-6}$
F22	$1.73\times 10^{-6}$	$2.58\times 10^{-3}$	$5.71\times 10^{-4}$	$3.88\times 10^{-6}$	$1.48\times 10^{-4}$
F23	$1.73\times 10^{-6}$	$1.73\times 10^{-6}$	$1.73\times 10^{-6}$	$1.73\times 10^{-6}$	$1.73\times 10^{-6}$
F24	$1.73\times 10^{-6}$	$2.60\times 10^{-6}$	$1.73\times 10^{-10}$	$1.73\times 10^{-10}$	$1.73\times 10^{-6}$
F25	$1.73\times 10^{-6}$	$8.94\times 10^{-4}$	$1.73\times 10^{-10}$	$1.02\times 10^{-5}$	$1.73\times 10^{-6}$
F26	$1.73\times 10^{-6}$	$1.36\times 10^{-5}$	$4.29\times 10^{-6}$	$2.35\times 10^{-10}$	$1.73\times 10^{-6}$
F27	$1.73\times 10^{-6}$	$5.71\times 10^{-2}$	$1.73\times 10^{-10}$	$1.73\times 10^{-10}$	$1.73\times 10^{-10}$
F28	$1.73\times 10^{-6}$	$6.32\times 10^{-5}$	$1.73\times 10^{-10}$	$2.84\times 10^{-5}$	$1.73\times 10^{-6}$
F29	$1.73\times 10^{-6}$	$3.88\times 10^{-6}$	$1.92\times 10^{-6}$	$1.73\times 10^{-6}$	$1.73\times 10^{-6}$
F30	$1.73\times 10^{-6}$	$1.92\times 10^{-6}$	$1.73\times 10^{-10}$	$1.73\times 10^{-10}$	$1.73\times 10^{-6}$

**Table 6:** Experimental results on the production optimization problem over 30 independent runs.

Algorithm	Mean (USD)	Std	Best	Worst	Wilcoxon (*p*)
GPS-CC-GME	$7.624\times 10^{8}$	$1.285\times 10^{7}$	$7.812\times 10^{8}$	$7.437\times 10^{8}$	—
GME	$7.215\times 10^{8}$	$2.147\times 10^{7}$	$7.528\times 10^{8}$	$6.924\times 10^{8}$	$2.8\times 10^{-5}$
BBO	$6.936\times 10^{8}$	$2.831\times 10^{7}$	$7.328\times 10^{8}$	$6.567\times 10^{8}$	$8.4\times 10^{-7}$
PO	$5.892\times 10^{8}$	$5.218\times 10^{7}$	$6.431\times 10^{8}$	$5.368\times 10^{8}$	$4.5\times 10^{-8}$
ESC	$7.382\times 10^{8}$	$1.893\times 10^{7}$	$7.654\times 10^{8}$	$7.125\times 10^{8}$	$5.2\times 10^{-4}$
DE	$6.794\times 10^{8}$	$3.152\times 10^{7}$	$7.185\times 10^{8}$	$6.426\times 10^{8}$	$2.7\times 10^{-7}$
PSO	$6.417\times 10^{8}$	$4.126\times 10^{7}$	$6.892\times 10^{8}$	$5.958\times 10^{8}$	$3.1\times 10^{-7}$
SMA	$6.153\times 10^{8}$	$4.735\times 10^{7}$	$6.674\times 10^{8}$	$5.645\times 10^{8}$	$1.2\times 10^{-7}$
CPA	$6.652\times 10^{8}$	$3.487\times 10^{7}$	$7.043\times 10^{8}$	$6.284\times 10^{8}$	$1.5\times 10^{-7}$
MGO	$7.073\times 10^{8}$	$2.486\times 10^{7}$	$7.421\times 10^{8}$	$6.741\times 10^{8}$	$5.6\times 10^{-6}$

## Data Availability

The original contributions presented in this study are included in the article material. Further inquiries can be directed to the corresponding author.

## Abbreviations

The following abbreviations are used in this manuscript:

GPS-CC-GME	Crisscross-Enhanced Groupers and Moray Eels Optimization
GME	Groupers and Moray Eels Optimization
HCS	Horizontal Crossover Search
VCS	Vertical Crossover Search
NPV	Net Present Value
BHP	Bottom-Hole Pressure
